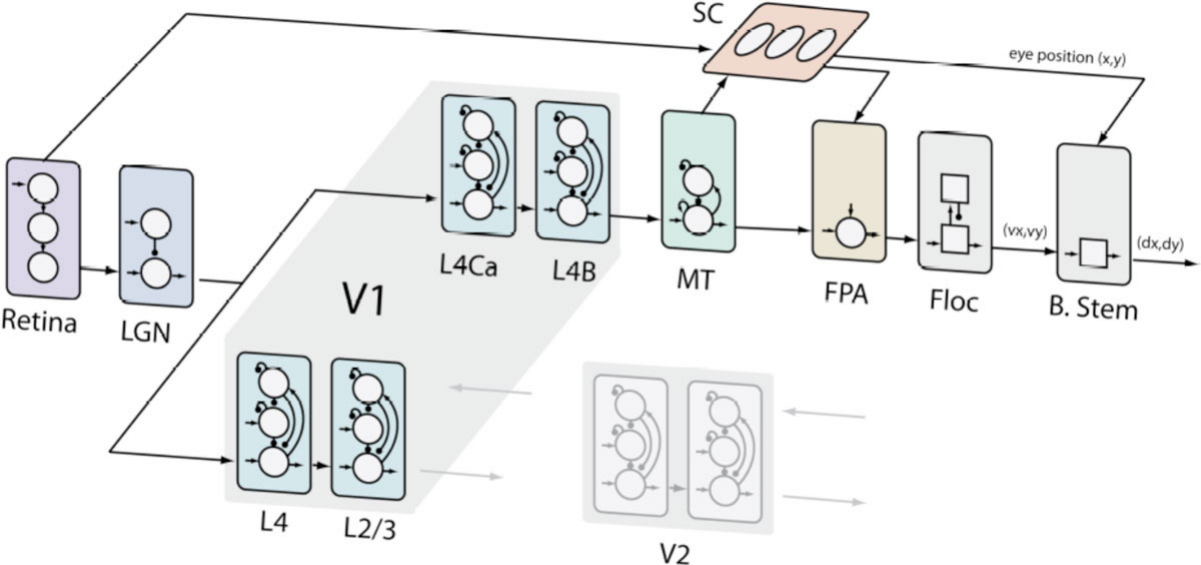# Spontaneous emergence of simple and complex receptive fields in a spiking model of V1

**DOI:** 10.1186/1471-2202-14-S1-O6

**Published:** 2013-07-08

**Authors:** Eugene M Izhikevich, Filip Piekniewski, Jayram Nageswaran, Csaba Petre, Micah Richert, Sach Sokol, Philip Meier, Marius Buibas, Dimitry Fisher, Botond Szatmary

**Affiliations:** 1 Brain Corporation, San Diego, California 92121, USA

## 

Brain Corporation is engaged in a multi-year project to build a spiking model of vision, paying special attention to the anatomy and physiology of the mammalian visual system. While it is relatively easy to hand-tune V1 to get simple and complex cells, it is not clear how to arrange connectivity in other cortical areas to get appropriate receptive fields, or what the appropriate receptive fields even should be. Instead of pre-wiring cortical connectivity according to a computational theory of how vision should work, we start with a generic "tabula rasa" spiking network having multiple cortical layers and neuronal types (single-compartment RS, FS, LTS cells). The goal is to find the anatomical and physiological parameters so that the appropriate connectivity emerges through STDP and visual experience. Since we know exactly what kind of receptive fields and visual responses are in V1, we build a smaller model of retina-LGN-V1 pathway and tune the STDP parameters so that the expected responses emerge. Admittedly, there are many free parameters that could be tuned to get V1-like responses; our choice is motivated by in-vitro recordings and other published data, whenever possible. Once we trust what we see in V1, we are ready to copy and paste the cortical model to implement V2, V3, V4, and IT areas with the hope that useful connectivity, receptive fields, and visual responses emerge. Our large-scale simulations of the spiking model of the visual system show spontaneous emergence of simple and complex cells, orientation domains, end-stopping receptive fields, extra-classical receptive fields with tuned surround suppression, color opponency that depends on the eccentricity of the receptive field, contrast invariance, and many other features that are routinely recorded in V1. Since the visual model exhibits micro- and full saccades, we observe perceptual behavior, such as the emergence of bottom-up (pop-out) attention. The model underscores the importance of spike-timing dynamics, inhibition, saccadic mechanism, and it imposes important restrictions on the possible types of STDP to model early visual processing.

This presentation is meant to be a flagship introduction to the whole effort by Brain Corporation to build spiking model of vision. There are multiple other submissions elaborating details of our modeling effort. The figure summarizes the gross anatomy of the spiking model of the visual system, including area MT, Superior Colliculus (SC), Frontal Pursuit Area (FPA), Flocculus (Floc), and Brainstem (B.Stem). The retina covers 10 by 10 degrees of visual field with the density of L, M, and S cones and receptive field sizes of Midget, Parasol, and SBC retino-ganglion cells corresponding to 4 degrees of eccentricity of a primate retina (collaboration with EJ Chichilnisky lab at Salk Institute). The model consists of more than 1M single-compartment neurons of RS, FS, and LTS types (Izhikevich, 2007 "Dynamical Systems in Neuroscience") and 0.25B synapses having AMPA, NMDA, GABA_A and GABA_B conductances, axonal conduction delays, and STDP. In this presentation, we summarize these and other anatomical and physiological assumptions, describe major results and point to other submissions by Brain Corporation scientists for more details.

**Figure 1:**